# Combining synchrotron X-ray diffraction, mechanistic modeling and machine learning for *in situ* subsurface temperature quantification during laser melting

**DOI:** 10.1107/S1600576723005198

**Published:** 2023-07-20

**Authors:** Rachel E. Lim, Tuhin Mukherjee, Chihpin Chuang, Thien Q. Phan, Tarasankar DebRoy, Darren C. Pagan

**Affiliations:** a Pennsylvania State University, University Park, PA 16802, USA; b Argonne National Laboratory, Lemont, IL 60439, USA; c Lawrence Livermore National Laboratory, Livermore, CA 14850, USA; Montanuniversität Leoben, Austria

**Keywords:** synchrotron X-ray diffraction, additive manufacturing, superalloys, laser melting, machine learning, heat-transfer and fluid-flow modeling, Gaussian process regression, temperature-distribution metrics, thermomechanical stress, elastic strains

## Abstract

Laser melting, such as that encountered during additive manufacturing, produces extreme gradients of temperature in both space and time, which in turn influence microstructural development in the material. Qualification and model validation of the process itself and the resulting material necessitate the ability to characterize these temperature fields. However, well established means to directly probe the material temperature below the surface of an alloy while it is being processed are limited. To address this gap in characterization capabilities, a novel means is presented to extract subsurface temperature-distribution metrics, with uncertainty, from *in situ* synchrotron X-ray diffraction measurements to provide quantitative temperature evolution data during laser melting. Temperature-distribution metrics are determined using Gaussian process regression supervised machine-learning surrogate models trained with a combination of mechanistic modeling (heat transfer and fluid flow) and X-ray diffraction simulation. The trained surrogate model uncertainties are found to range from 5 to 15% depending on the metric and current temperature. The surrogate models are then applied to experimental data to extract temperature metrics from an Inconel 625 nickel superalloy wall specimen during laser melting. The maximum temperatures of the solid phase in the diffraction volume through melting and cooling are found to reach the solidus temperature as expected, with the mean and minimum temperatures found to be several hundred degrees less. The extracted temperature metrics near melting are determined to be more accurate because of the lower relative levels of mechanical elastic strains. However, uncertainties for temperature metrics during cooling are increased due to the effects of thermomechanical stress.

## Abstract 2

A new methodology is demonstrated for analyzing spatial temperature distributions using modeling and machine learning. The method is applied to X-ray data gathered during *in situ* laser melting.

## 1. Introduction

Primary factors for controlling the microstructure, porosity and residual stress state during additive manufacture (AM) of engineering alloys include heat input, the resulting temperature and temperature gradients through the component. Heat treating of components using the heating sources themselves is also of increasing importance. In response, significant efforts have been undertaken to characterize temperature profiles during AM builds in order to guide the build-design process. These efforts include both thermal and optical imaging of the specimen surfaces during a build to estimate both surface temperature and melt-pool shape (Moylan *et al.*, 2014 ▸; Fox *et al.*, 2017 ▸; Fisher *et al.*, 2018 ▸; Montazeri *et al.*, 2019 ▸; Dunbar & Nassar, 2018 ▸; Forien *et al.*, 2020 ▸; Ashby *et al.*, 2022 ▸), along with predicting defect formation. While valuable, these characterization efforts only provide information about the temperature profile at the sample surface, precluding understanding critical subsurface thermal profiles during initial melting and subsequent reheating events as layers are added above a volume of material of interest. Rather, different measurement modalities are needed to probe an alloy’s evolving microstructure during the repeated thermal cycling encountered during the build process.

To address these challenges, new synchrotron X-ray imaging and diffraction capabilities have been developed that can characterize structure at rapid timescales (≪ s). These measurements probe the subsurface thermomechanical state (thermal and mechanical lattice strain) and microstructure evolution in conditions mimicking a wide range of additive-manufacturing processes (Kenel *et al.*, 2016 ▸; Calta *et al.*, 2018 ▸; Cunningham *et al.*, 2019 ▸; Hocine *et al.*, 2020 ▸; Oh *et al.*, 2021*a* ▸,*b* ▸; Thampy *et al.*, 2020 ▸). During these experiments, average temperatures of the crystalline phases within a diffraction volume are estimated from the shifts in diffraction-peak centroid positions due to a convolution of microstructure evolution and thermal and mechanical strains. The effects of stress (elastic strains) and microstructure evolution (such as changes in local composition or precipitation) are often neglected. With knowledge of the coefficient of thermal expansion (CTE) of the material within the applicable range of temperatures, and assuming equilibrium CTEs are valid during rapid cooling, thermal strains are mapped directly to temperatures. While this peak centroid analysis is valuable, the accuracy of the temperatures obtained can be compromised due to the inherent spatial gradients of temperature, mechanical loading and chemistry during the build process. However, while a complication for data analysis, information regarding the spatial gradients of temperature (along with mechanical strains and chemistry variation) within a diffraction volume is encoded into each diffraction peak. Unfortunately, the single projection of X-rays through the diffraction volume during these experiments prevents the direct inversion or reconstruction of the temperature field in a tomographic fashion.

Here, we propose a novel path forward to extracting these temperature data, in which a mechanistic heat-transfer and fluid-flow model and X-ray simulations provide a framework for interpreting and analyzing complex experimental temperature distributions. The simulations are used to create a collection of reference diffraction patterns (a ‘dictionary’) representing different thermal states. A strategy is then adapted from Bamney *et al.* (2020 ▸) to use Gaussian process regression (GPR) to learn mappings between spatial temperature distribution metrics within a diffraction volume (generated from mechanistic modeling) and diffraction peak shapes. The GPR approach taken here is a transfer learning process in which GPR is applied to simulated training data sets, and then the ‘learned’ relationships between spatial temperature distribution metrics within a diffraction volume and diffraction peak shapes are transferred to experimental data sets of interest. The simulation includes both heat-transfer and fluid-flow modeling coupled with X-ray diffraction modeling to generate synthetic diffraction data sets. The accuracy of GPR processes as used here depends on how well they can predict the temperature-field ‘outputs’ (descriptors of the temperature fields present) given particular diffraction ‘inputs’ (diffraction data). Since training is performed with synthetic data sets, the temperature fields used to create the diffraction data sets are known. As such, the accuracy of the diffraction modeling is more critical than the heat-transfer and fluid-flow modeling (although if the heat-transfer and fluid-flow modeling is accurate, uncertainties will be decreased). Fortunately, the physics of X-ray diffraction are well understood. When applying the trained GPR models to experimental data, the uncertainties reflect differences between the training and testing data.

## 2. Material and experiment description

**Figure 1:**
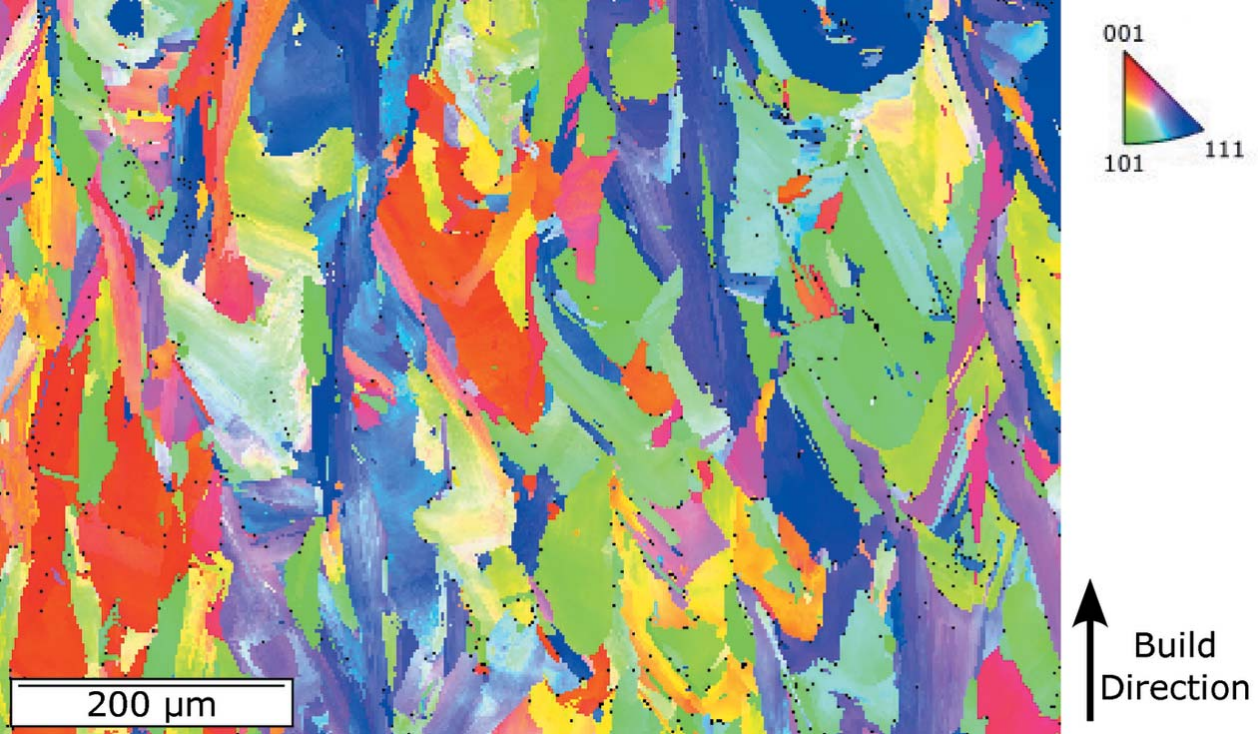
A representative orientation map measured using electron backscatter diffraction from the thin-wall specimen tested in this work. Crystal directions are colored with respect to the build direction in the provided inverse-pole-figure color map.

The specimens in both experiment and simulations used in this work were AM Inconel 625 (IN625) nickel superalloy made using laser powder bed fusion (LPBF) at the National Institute of Standards and Technology (NIST), designation PBF-LB-IN625. The experimental specimen was a thin wall 3 mm in height along the build direction ( 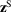 ) by 0.53 mm in thickness ( 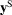 ) and 20 mm in length ( 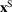 ). The experimental specimen was extracted using electro-discharge machining from a block (50 × 15 × 6 mm) built in an EOS M290 machine using manufacturer-recommended build parameters (Son *et al.*, 2020 ▸). The IN625 powder used for the build was obtained from the machine manufacturer EOS. The build layer thickness was 40 µm, with a 110 µm hatch spacing. The laser power and speed were 285 W and 960 mm s^−1^, respectively. The interlayer rotation during the build of the larger block from which the thin-wall specimen was extracted was 67.5°. After the build and prior to the thin-wall specimen extraction, the block was stress-relief heat treated at 1073 K for 2 h. The IN625 used in this work was built using the same machine, powders and build parameters of material utilized for the NIST AM Bench 2018 challenge (Levine *et al.*, 2020 ▸). An orientation map measured using electron backscatter diffraction from the thin-wall specimen prior to laser remelting is shown in Fig. 1 ▸. Crystal directions are colored with respect to the build direction using an inverse-pole-figure color map. The microstructure primarily consists of large grains with dimensions of the order of 100 µm, interspersed with smaller grains of the order of 10 µm.

**Figure 2:**
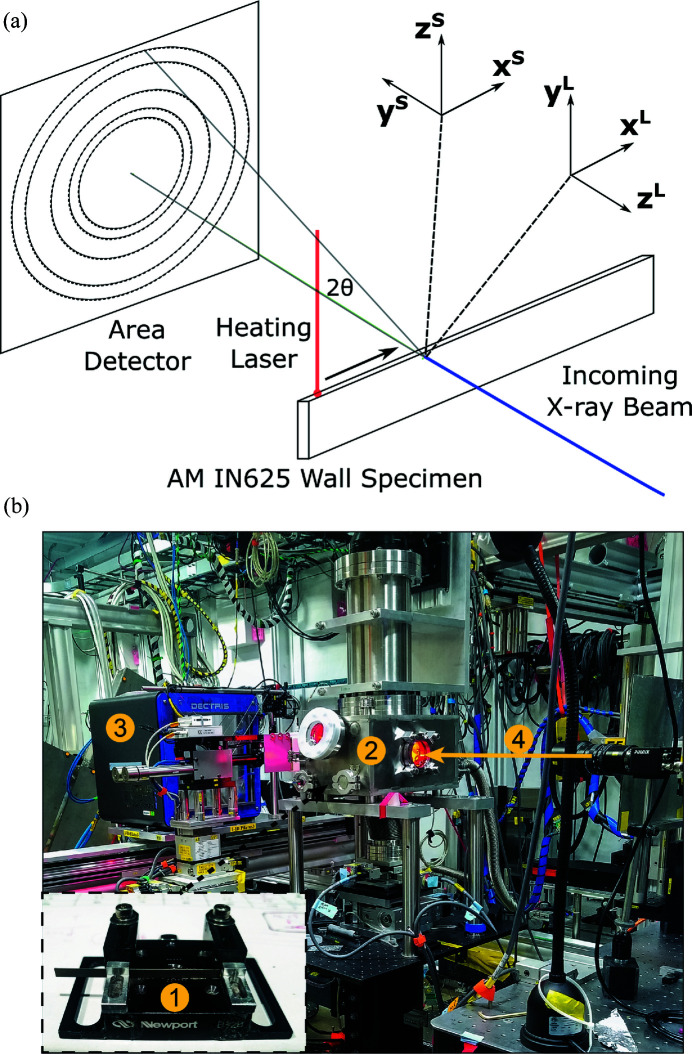
(*a*) A schematic diagram of the experimental setup with ‘S’ superscripts for the sample reference frame associated with the heat-transfer and fluid-flow modeling and ‘L’ superscripts for the laboratory reference frame associated with the laser heating and with the X-ray diffraction simulations. (*b*) A photograph of the experimental measurement setup used to collect X-ray diffraction data for developing the temperature-extraction framework. Marked are (1) the sample holder and sample, (2) the laser test system, (3) the X-ray area detector, and (4) the incoming beam direction.

*In situ* diffraction measurements during laser melting were performed at beamline 1-ID at the Advanced Photon Source (APS). Fig. 2 ▸(*a*) shows a schematic diagram of the experimental geometry for the measurements including the AM IN625 wall specimen, heating laser, incoming X-ray beam, and orientations of the sample (S) and laboratory (L) coordinate systems. In the laboratory coordinate system, the incoming X-ray beam travels in the 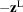 direction while the heating laser was nominally aligned along 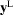 . During X-ray measurements, the specimen remained fixed in the laboratory coordinate system as the laser traveled in the 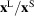 direction. The angle between incoming and diffracted X-rays, 2θ, is labeled and is related to the spacing of diffracting sets of lattice planes. The incoming X-ray beam was 61.332 keV and was focused vertically by a set of Si sawtooth lenses to dimensions of 50 × 30 µm along 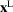 and 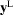 , respectively. X-ray diffraction images were measured by a Pilatus3 X CdTe 2M detector sitting 752 mm downstream of the sample. The detector has 1475 × 1679 pixels and a pixel size of 172 × 172 µm. Diffraction images were collected with an exposure time of 1 ms and a frequency of 250 Hz throughout the experiment.

**Figure 3:**
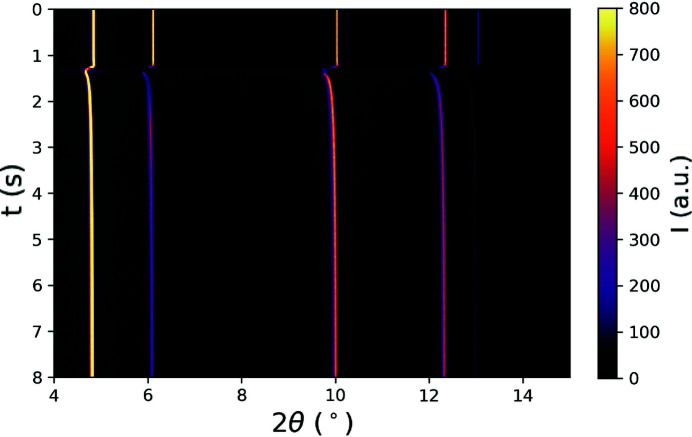
Evolution of azimuthally integrated diffracted intensity *I* with time *t* during laser melting of the IN625 wall specimen. Each row corresponds to the diffraction line profile (intensity *I* versus Bragg angle 2θ) for a given time step, with the color signifying the magnitude of diffracted intensity.

Laser melting was performed using an existing *in situ* LPBF simulator at the APS. A picture of the simulator in Sector 1-ID is shown in Fig. 2 ▸(*b*) and more complete system details are provided by Zhao *et al.* (2017 ▸). The LBPF simulator uses an ytterbium fiber laser (IPG YLR-500-AC, USA) and an intelliSCAN_de_ 30 for laser motion. Prior to laser melting, the environment chamber was purged and re-filled with high-purity argon gas. During testing, the specimen was placed 2.9 mm away from the laser focal plane to create a spot diameter of 100 µm. During X-ray diffraction measurements, the laser was rastered over the wall specimen along 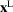 with a laser power of ∼120 W and a speed of 0.05 ms^−1^. This linear power density (2400 J m^−1^) is relatively high in comparison with standard LPBF parameters for IN625. These parameters were chosen to ensure a relatively large melt pool and extended temperature gradient through the thickness of the specimen. Fig. 3 ▸ shows the diffracted intensity integrated azimuthally around the detector (along the diffraction rings) versus time. In the figure, we can see the shifting of the diffracted intensity to lower 2θ at 125 ms. This shift to lower 2θ is due to an increase in lattice plane spacing as the diffraction volume is rapidly heated because of the laser passing over it.

## 3. Methods

**Figure 4:**
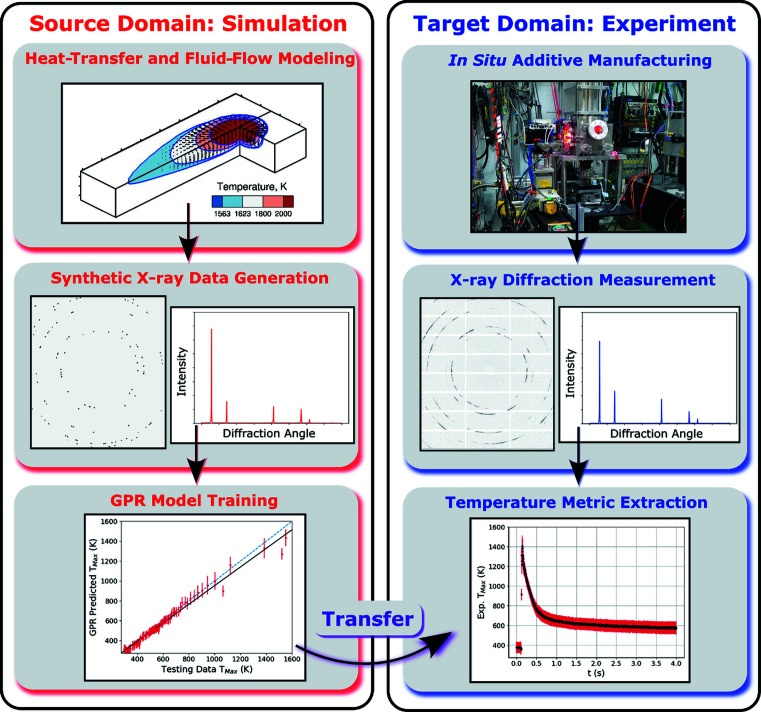
A flow chart showing the steps included in this work to extract temperature metrics from X-ray diffraction data. Within the source domain, heat-transfer and fluid-flow modeling is used to inform X-ray diffraction simulations, which are then used to train GPR models. The trained GPR models are then transferred to the target domain and used to predict temperature metrics from the X-ray experimental data.

In this section, an overview of the various methods employed to build the dictionary of reference diffraction patterns linked to underlying thermal distributions is given. A description of the GPR model, training, and hyperparameter selection used to learn the mapping between underlying temperature distribution metrics and diffraction line profiles is also provided. As previously described in the *Introduction* [Sec sec1], as opposed to using X-ray diffraction data to validate a simulation, we are using combinations of heat-transfer and fluid-flow modeling, X-ray diffraction simulation, and machine learning to interpret and extract information from the experimental data. A schematic diagram of the various components of our method is shown in Fig. 4 ▸, and the components of the modeling and training parts are described in more detail in the following subsections. As a short summary, synthetic X-ray data are generated using thermal fields output from the mechanistic heat-transfer and fluid-flow model. We adopt an approach outlined in Fig. 4 ▸ in which pairs of synthetic X-ray line profiles (input) and underlying temperature metrics (output) are used to train various GPR surrogate models. Then, the surrogate models, trained using synthetic data (the source domain), are ‘transferred’ (Weiss *et al.*, 2016 ▸) to analyze experimental data (the target domain) to determine temperature metrics through, essentially, comparisons with the previously generated synthetic data.

### 3.1. Heat-transfer and fluid-flow modeling

A heat-transfer and fluid-flow model was utilized to calculate the 3D transient temperature and velocity fields during the laser melting of IN625 specimens. The model is discussed in detail by Mukherjee *et al.* (2018*a* ▸,*b* ▸) and only the important features are described here. It was developed using in-house Fortran code compiled with an Intel Compiler. The model calculates the melt-pool size, temperature fields and velocity fields during the LPBF process, taking the laser parameters and alloy and environmental gas properties as inputs. The model considers temperature-dependent thermophysical properties for both powder and fully dense material. The thermophysical properties of IN625 required in the model were calculated using the commercial package *JMatPro* (https://www.sentesoftware.co.uk/jmatpro), while the uncertain parameters such as absorptivity and the power distribution of the laser beam can be adjusted to match the experimental data on thermal cycles and deposit geometry. The material parameters used for the heat-transfer and fluid-flow modeling are provided in Table 1 ▸. The model iteratively solves the equations of conservation of energy, mass and momentum in a 3D computational domain consisting of the substrate, power bed, deposited layers and hatches, and shielding gas. The equations are discretized in the computational domain using a finite difference scheme, and a traveling grid approach is used to increase computational efficiency. The model provides accurate results on melt-pool geometry and temperature fields by considering the effects of the convective flow of molten metals. While laser melting and the resulting temperature distributions within solid specimens are modeled in this work, the same model is capable of modeling temperature distributions within loose powder layers (Mukherjee *et al.*, 2018*a* ▸,*b* ▸).

Using this model, a series of single laser trace simulations were performed around conditions similar to the previously performed *in situ* synchrotron experiments (Section 4.2). For all simulations, a unidirectional scan along 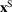 of the laser beam was used, with the laser-beam direction being 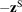 . Positive 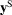 is perpendicular to the laser scanning direction and represents the direction along the width of the wall specimen. The laser power and velocity were varied around the nominal experimental parameters (120 W laser power and 50 mm s^−1^ laser speed). In total, nine laser-melting simulations were performed according to the simulation test matrix in Table 2 ▸. Each simulation captured the laser traveling over a 200 ms interval with 200 µs time steps while temperature fields were output every 2 ms. The heat-transfer and fluid-flow simulations were performed on the ROAR supercomputer at Pennsylvania State University using 40 cores (2.13 GHz) for each simulation, with each thermal simulation taking 1 h to complete.

### 3.2. Synthetic X-ray data generation

Each of the nine laser trace simulations were used to generate a series of time-dependent area-detector X-ray diffraction patterns using the framework developed by Pagan *et al.* (2020 ▸). This simulator uses diffraction calculation and projection algorithms contained in the Python-based *HEXRD* software package (Bernier *et al.*, 2011 ▸). In the diffraction-simulation framework, X-ray diffraction is simulated within a polychromatic (Laue) diffraction framework that employs a finite-energy bandwidth to capture realistic beam conditions. This method provides benefits over angle-based diffraction solution methods in conditions where the specimen is not rotating, such as the single laser-trace experiments described above. Here, the average X-ray energy and bandwidth (|Δ*E*|/*E*) were chosen to match the experiment, 61.332 keV and 5 × 10^−4^, respectively.

In this framework, diffraction events are simulated from discretized volumes in space, with the spatial positions of each volume being incorporated into diffracted ray-tracing calculations. For clarity, we will refer to these discretized volumes as scattering volumes, while the total volume illuminated by the X-ray beam is the diffraction volume. Inside each scattering volume, individual grains (lattice orientations) are inserted from which diffraction events are calculated. In these diffraction simulations, the grains have no morphological features and their orientations are randomly generated. The thermomechanical state of each scattering volume, and the accompanying changes in lattice state, can vary spatially. Here, temperature fields produce spatially varying thermal strain within the diffraction volume. Matching the experiment, the simulated diffraction volume is 50 × 30 × 530 µm, while each scattering volume was 20 × 20 × 20 µm. Each scattering volume contained two randomly oriented grains simulating grains with ∼25 µm equivalent diameter. Each grain contains 1° of lattice misorientation to provide some diffraction peak broadening.

To model the thermal strain’s effect on the measured diffraction data, the lattice structure of an embedded grain is altered by stretching the reciprocal lattice vectors, 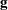 , of the grain isotropically (valid for a cubic crystal): 

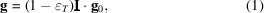

where 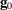 is the unstrained reciprocal lattice vector in a crystal, 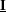 is the second-order identity tensor and the thermal strain ɛ_*T*_ is given by 

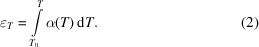

**Figure 5:**
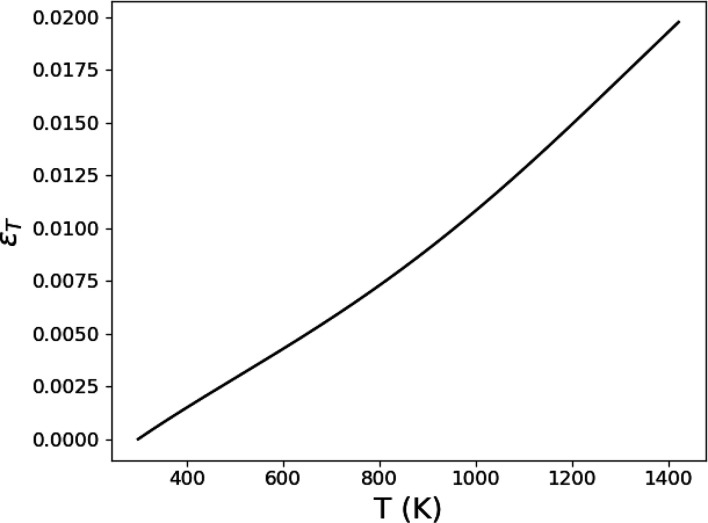
Measured thermal strain ɛ_*T*_ versus temperature *T* of IN625 used for the diffraction simulations. Measurements were made using dilatometry on the same material as tested in the synchrotron experiment.

The temperature-dependent CTE α(*T*) used in this work was measured independently using bulk dilatometry measurements at the Center for Innovative Sintered Products, Pennsylvania State University. Bear in mind, these measurements describe equilibrium thermal-expansion behavior, which may not exactly capture thermal expansion during rapid heating and cooling. The sample used for dilatometry measurements was extracted via electro-discharge machining from an identically built AM bulk sample to that used for the *in situ* diffraction experiments. The thermal strain ɛ_*T*_ as a function of temperature determined from these measurements and used for diffraction simulations is provided in Fig. 5 ▸. The reference lattice parameter from which 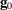 were generated was 3.5981 Å. As only crystalline material will diffract, if the temperature in a scattering volume exceeds the solidus temperature (1563 K; Pawel & Williams, 1985 ▸), no diffraction events are recorded. For the high-energy transmission geometry used in the experiment, all diffracting X-rays have nearly the same path length regardless of whether they diffract from the upstream or downstream side of the specimen. As such, absorption is not considered since it will only scale the total integrated intensity of the diffraction peak and not change the peak shape.

**Figure 6:**
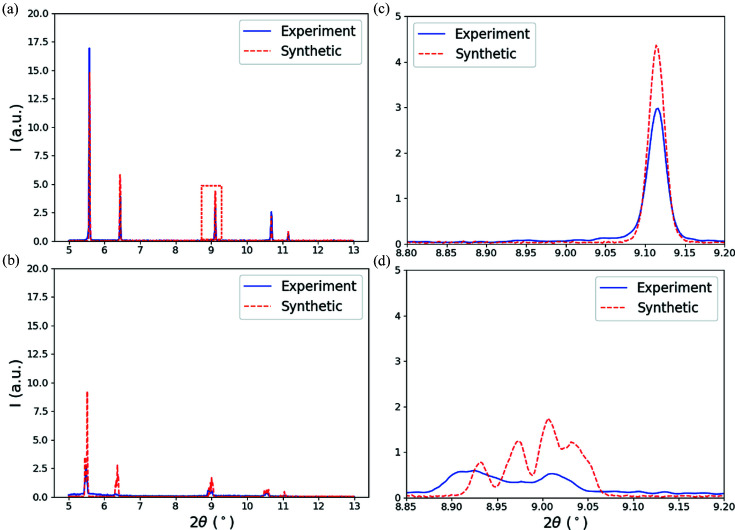
Comparison between experimental (blue) and synthetic (dashed red) diffraction line profiles (*I* versus 2θ) from the AM IN625 wall specimens in representative (*a*) unheated and (*b*) heated conditions. Enlarged views of the 220 diffraction peak in the (*c*) unheated and (*d*) heated conditions. The 220 peak provides an example of relatively extreme peak splitting due to the temperature gradient present.

The laser-trace simulations described in Section 3.1 were used as input for generating synthetic diffraction images. As the thermal simulations use a traveling grid formulation for calculation, thermal fields at each time step were mapped to a regular grid of scattering volumes with 20 µm spacing in all three directions. Each thermal simulation was used to generate three sets of synthetic diffraction data capturing the evolution of different temperature gradients by placing the X-ray beam at different portions of the sample. For each thermal-simulation time series, the center of the X-ray beam was placed 20, 40 and 60 µm below the top of the specimen, and separate 2D X-ray diffraction image sets were generated. In total, 27 sets of X-ray simulations and 2700 diffraction images were generated for surrogate model training. Once the 2D diffraction patterns were simulated for the entire time series, each pattern was integrated azimuthally around the diffraction rings to create 1D diffraction line profiles (intensity versus 2θ). The integrated diffraction line profile data cover 2θ angles ranging from 5 to 13° and encompass the first six sets of lattice planes: (111), (200), (220), (311), (222) and (400). After integration, background noise corresponding to scattering in the experimental station was added to the synthetic data. A comparison of example experimental (blue) and synthetic (dashed red) diffraction line profiles in the unheated conditions is given in Fig. 6 ▸(*a*) and soon after laser heating in Fig. 6 ▸(*b*). Differences in relative peak heights are probably due to local texture in the thin-wall sample. Figs. 6 ▸(*c*) and 6 ▸(*d*) show enlarged views of the 220 diffraction peak in the unheated and heated conditions, respectively. Relatively extreme peak broadening and splitting due the temperature gradient present can be observed in both the experimental and synthetic diffraction images. However, the goal of the diffraction simulations is not to exactly match the experimental diffraction line profiles but to provide a reference dictionary that a trained GPR surrogate model can utilize to predict a temperature metric based on ‘similar’ features found in the data of interest. Each diffraction simulation of a heating time series at a single beam position took ∼4 h to complete, with diffraction calculations from each scattering volume parallelized over 40 cores.

### 3.3. GPR surrogate model description

**Figure 7:**
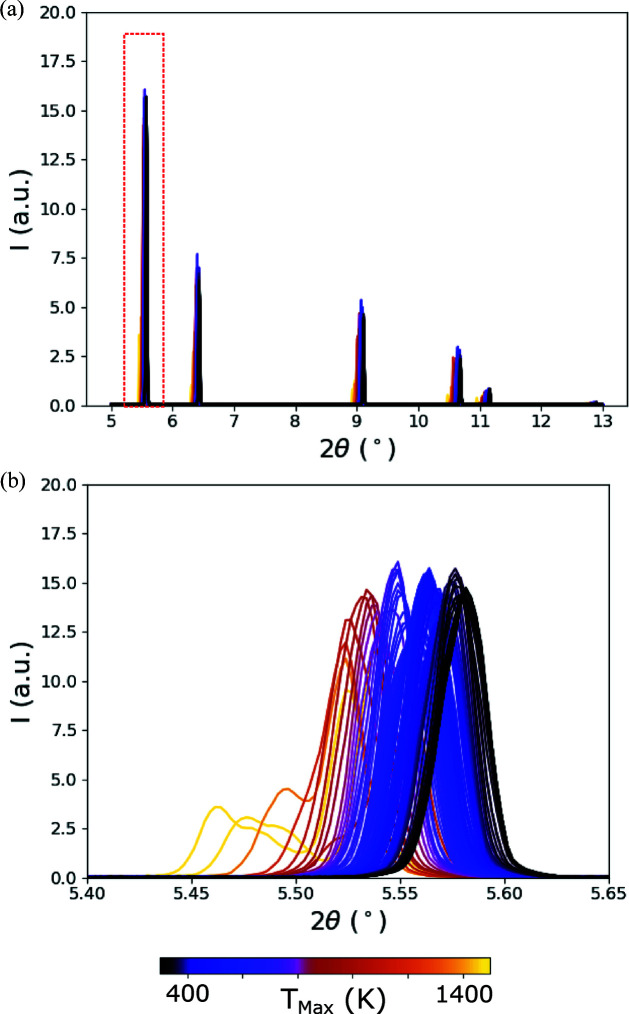
Simulated (*a*) diffraction line profiles (*I* versus 2θ) and (*b*) 111 diffraction peaks of IN625 colored by the maximum temperature (*T*_Max_) in the diffraction volume which was used to generate the pattern.

Here we utilize a supervised machine-learning technique, GPR (Rasmussen & Williams, 2006 ▸) implemented via *scikit-learn* (Pedregosa *et al.*, 2011 ▸), to learn mapping between diffraction line profiles and various temperature metrics in the diffraction volume. Fig. 7 ▸ shows example synthetic diffraction patterns generated using the mechanistic AM and X-ray diffraction modeling colored by a temperature metric of interest in the diffraction volume (maximum temperature, *T*_Max_). The goal of using GPR is to learn these mappings between line profiles and underlying thermal distributions such that, with a diffraction line profile, a temperature metric of interest can be extracted. Again, this places emphasis on the accuracy of the X-ray diffraction simulation, rather than the mechanistic heat-transfer and fluid-flow modeling. The method was recently applied to developing a mapping between diffraction line profiles and dislocation configurations within diffraction volumes (Bamney *et al.*, 2020 ▸). In addition, the GPR approach shares commonalities with the Bayesian Rietveld approach introduced by Ida & Izumi (2011 ▸).

GPR takes a Bayesian statistical approach to surrogate model prediction. The GPR method creates a normal distribution of mapping functions, informed by training data, with the mean of function distribution serving as a model prediction. The variance of the function distribution can serve as a confidence bound or to inform where more training data may be necessary (*i.e.* where there is high variance). In GPR, model output predictions (*i.e.* temperature metrics) are constructed from linear combinations of transformations of the input data (*i.e.* diffraction line profile data). The transformation and linear weights are fitted according to the input training data and a chosen covariance (kernel) function. In general, the variance for a given prediction reflects the difference between the GPR model input and training data used to build the model. For example, GPR input (*i.e.* a diffraction line profile) that exactly matches the training data will have a variance of zero, while input that is very different from the training data (*e.g.* a new phase is present) will produce a prediction (*i.e.* a temperature metric) with a very high variance. As the training data become more accurate (or at least closer to the model input of interest), the variance or uncertainty is reduced.

The most common kernel function, *k*, in GPR is the Gaussian kernel (also often referred to as the squared exponential kernel, exponentiated quadratic kernal or radial basis function kernel). The kernel dictates the weights used to make predictions from training-data points. The Gaussian kernel takes the form 

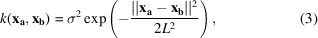

where 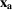 and 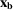 are input data vectors, and σ^2^ is the amplitude. For this work, the input data vectors are intensity values in diffraction line profiles. This kernel includes a length scale, *L*, which controls the extent of influence of a data point, affecting the variance of the function distribution. The Gaussian kernel is referenced because of its representative behavior and familiarity with the shape of Gaussian functions. As the distance between an input vector ( 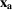 ) and a data point ( 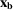 ) is decreased, the weight increases. Conversely, as the distance increases the weights decay in an exponential fashion.

Here, we employ the related rational quadratic kernel, which is equivalent to the summation of many exponentiated quadratic kernels of different length scales: 

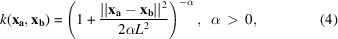

where α is the relative weighting between large and small length scales. Accordingly, increasing α reduces the amount of local variation (slows the weighting decay rate), and, when α → ∞, the rational quadratic kernel converges to the exponentiated quadratic kernel. A range of *L* and α values were tested for model training, but the closest fits to the training data (without overfitting) corresponded to *L* = 1 and α = 1.

### 3.4. GPR model training and temperature-metric extraction

Prior to application of the GPR models to the experimental synchrotron data, the accuracy of GPR predictions was evaluated using a set of reserved simulations. GPR models were trained using 26 of the 27 synthetic diffraction data sets, comprising 2600 images (again, the nine laser parameter sets given in Table 2 ▸ with three beam positions each), using a single processor. The reserved synthetic diffraction time series data correspond to conditions best matching the experiment: 120 W laser power, 0.05 ms^−1^ laser speed and placing the X-ray beam 20 µm below the top of the sample. After GPR surrogate model training and testing of the models against reserved simulated data, the trained GPR models were applied to the X-ray diffraction data collected during the synchrotron experiment. In this process, the diffraction data collected at each time step are treated as an independent data point and used as input for the various GPR models. At the end of the process, various temperature-metric histories for the diffraction volume probed during the experiment are generated.

## 4. Results

### 4.1. Surrogate model training

**Figure 8:**
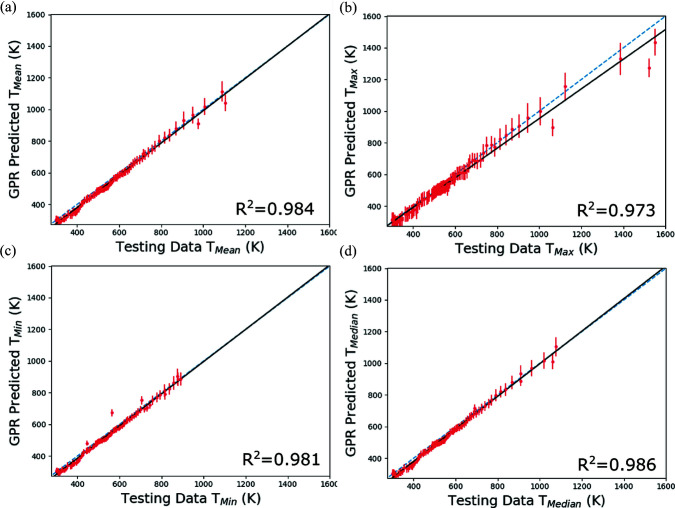
Comparison of prediction of temperature metrics from trained GPR surrogate models using simulated diffraction data input reserved from model training. The metrics are the (*a*) mean *T*_Mean_, (*b*) maximum *T*_Max_, (*c*) minimum *T*_Min_ and (*d*) median *T*_Median_ of the temperature distribution present in the diffraction volume. The red error bars correspond to the square root of the variance (standard deviation) of the GPR predictions. A linear regression line has been fitted to the testing data and GPR predictions and is shown with a black line. The dashed blue line corresponds to perfect correlation between reserved testing data and GPR model predictions.

Fig. 8 ▸ shows predictions of four temperature metrics within the diffraction volume using trained GPR models (each metric has its own trained GPR model) that use diffraction line profiles as input compared with the same temperature metrics extracted directly from the reserved thermal simulations. The four temperature metrics are the mean *T*_Mean_ [Fig. 8 ▸(*a*)], maximum *T*_Max_ [Fig. 8 ▸(*b*)], minimum *T*_Min_ [Fig. 8 ▸(*c*)] and median *T*_Median_ [Fig. 8 ▸(*d*)] temperatures. As previously described, for each GPR surrogate model prediction, the variance of the prediction associated with the normal distribution of the mapping functions can also be calculated and employed as an uncertainty. In Fig. 8 ▸, the uncertainty (square root of the variance, standard deviation) of the GPR prediction is shown by the red error bars. For each temperature metric, a linear regression line was fitted to the GPR predictions and is plotted with a black line. A blue dashed line corresponding to perfect correlation between the GPR model predictions and the reserved testing data is provided for comparison. The *R* ^2^ coefficient of determination of the GPR predictions is also provided.

As a whole, there is very good agreement between the reserved testing data and the GPR model predictions. No aphysical predictions are found across the metrics, such as predictions of temperature significantly below room temperature or above the solidus temperature. There are generally more training and testing data in the lower-temperature regions due to the laser passing rapidly over the specimen, leading to generally increased uncertainty at higher temperatures (see the larger red error bars). This feature is most notable for the maximum-temperature predictions near the solidus temperature, which have the largest uncertainties (of the order of 200 K or 15%). Conversely, the mean-temperature predictions have the smallest uncertainties (of the order of 20–40 K or 6–12%), which is not surprising. The mean temperature is strongly correlated to the point of highest intensity on the diffraction peak. This can be contrasted with the minimum and maximum temperatures, which generally correspond to small volumes of material contributing to the tails of the diffraction peaks. Again, these temperature-metric predictions take into account contributions to peak broadening from the spatial distribution of temperature within the diffraction volume. In other words, the hottest and coldest regions may not necessarily correspond to the most extreme tail positions on the diffraction peak depending on the spatial location of the diffraction event.

### 4.2. Application of GPR surrogate models to experimental data

**Figure 9:**
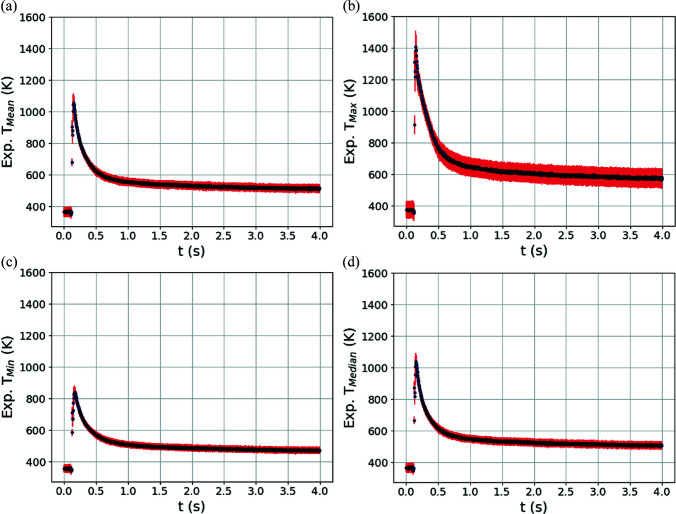
The evolving (*a*) mean *T*_Mean_, (*b*) maximum *T*_Max_, (*c*) minimum *T*_Min_ and (*d*) median *T*_Median_ of the distribution of temperature within the experimental X-ray diffraction volume with respect to time *t*, extracted using the trained GPR surrogate models. The red error bars correspond to the square root of the variance (standard deviation) of the GPR surrogate model predictions.

We have trained a series of GPR surrogate models for predicting temperature metrics from synthetic diffraction line profiles. Here we apply the trained models to analyzing experimental data captured during the synchrotron experiment described in Section 2. Fig. 9 ▸ shows the evolution of the mean *T*_Mean_ [Fig. 9 ▸(*a*)], maximum *T*_Max_ [Fig. 9 ▸(*b*)], minimum *T*_Min_ (Fig. 9 ▸(*c*)] and median *T*_Median_ [Fig. 9 ▸(*d*)] temperatures within the diffraction volume versus time. Again, the red error bars associated with each temperature-metric measurement correspond to the uncertainty in the extracted quantity as given by the square root of the variance of the GPR prediction.

The point where the moving laser passes over the diffraction volume is the clear peak in all four metrics. At its highest point, the prediction for *T*_Max_ is close to the solidus temperature for IN625, as expected, since melted material will not contribute to the diffraction peaks. Similarly to the cross validation with the simulated data, *T*_Max_ has the highest uncertainty (largest error bars). We also observe across all four metrics that the temperature remains relatively high well after the laser has passed over the diffraction volume (of the order of 200 K above room temperature). As will be discussed, this may be due to thermomechanical stress developing within the specimen upon rapid cooling, as a positive mean stress in the volume probed will appear as an elevated temperature. As previously described, the uncertainty in the temperature predictions is related to how close input diffraction line profiles are to data used for GPR model training. With the transfer learning approach, if the simulations in the source domain used to generate the training data are missing physics, such as the development of stress due to thermal gradients, the accuracy in the target domain will decrease. Taking this into account, temperature-metric values closer to the solidus temperature are probably the most accurate, since at this point thermal expansion is at its largest and the mechanical stresses are at their lowest. This will be further discussed in Section 5.2.

## 5. Discussion

Here, we have described and demonstrated a novel approach for quantifying temperature distributions within alloys during extreme heating processes that utilizes *in situ* synchrotron X-ray diffraction, mechanistic modeling and X-ray simulation, and supervised machine learning. The approach was applied to quantifying temperature metrics in the bulk of an IN625 specimen during high-speed laser melting mimicking AM. The development of approaches such as that presented in this article is important for quantifying and controlling temperature distributions during the AM build process. In turn, this information can be used to accelerate process certification and optimization of build routines to control microstructure and minimize defects. The presented method is a significant advance from current X-ray diffraction based approaches in the literature that are only capable of estimating the average temperature in an illuminated volume (Hocine *et al.*, 2020 ▸; Oh *et al.*, 2021*a* ▸,*b* ▸). Usually, analytical functions (*e.g.* Gaussian or Lorentzian) are fitted to the diffraction line profiles and the temperature is calculated from shifts of diffraction peaks. Often, to improve temperature extraction from peak fittings, experiments are conducted such that melt pools are much larger and scanning conditions are unrealistic. During this process, numerous assumptions are made regarding the temperature distribution present and X-ray interaction with the sample, which increases the uncertainty of the estimation, most notably that all X-rays are emitted from a point source and the shape of the function used to fit the diffraction peak. In addition to decreasing accuracy with these assumptions, all information about the temperature distribution in the illuminated volume is lost.

Our effort addresses these shortcomings by directly accounting for realistic spatial thermal gradients. With these gradients accounted for, temperature metrics determined from experimental data will have increased accuracy, and we can access information about thermal gradients, a major driver of microstructure formation. Now we will examine the temperature distribution that was probed during the *in situ* measurements. While the method presented is a major advance forward for bulk temperature quantification during AM, we will also discuss means to further increase accuracy and to extend the method to other quantities, such as melt-pool volume.

### 5.1. Temperature-distribution evolution

**Figure 10:**
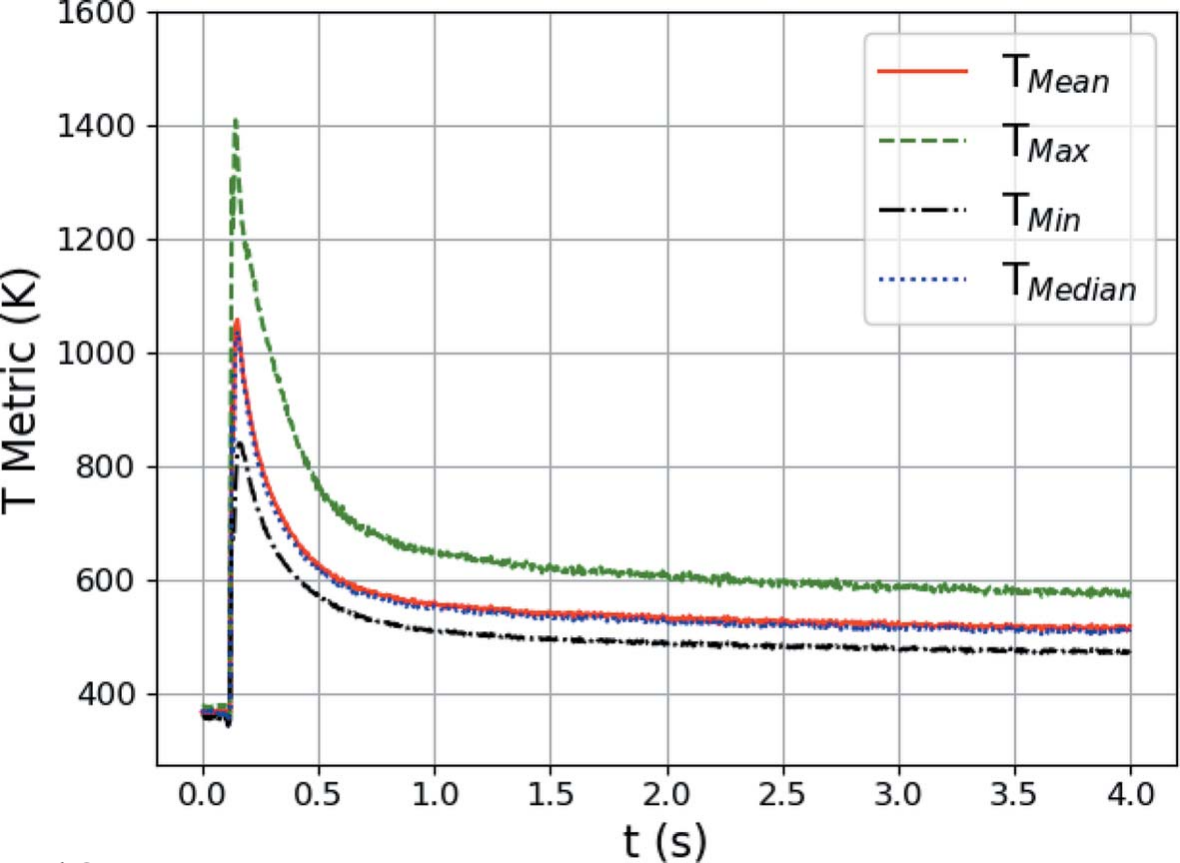
Comparison of the evolution of temperature *T* metrics with time *t*, extracted from the experimental diffraction data using the GPR surrogate models.

A primary benefit of the approach presented is the ability to explore the evolving distributions of temperature present within the diffraction volume. As an example, we can analyze the distribution of temperature within the diffraction volume during the *in situ* synchrotron experiment. Fig. 10 ▸ shows the evolution of the mean, maximum, minimum and median temperature metrics extracted using the various GPR surrogate models together. With regards to the temperature distribution, of most interest is the range (difference of maximum and minimum) of temperatures in the crystalline phase throughout the diffraction volume. From the figure, we can see that there is nearly a 600 K difference between the maximum and minimum temperatures in the solid phase as the laser passes over the diffraction volume. We can also see that the temperature difference remains throughout cooling and is still over 100 K at the end of the measurement. As the mean and median temperatures are closer to the minimum temperature than the maximum, we can infer that the bulk of the diffraction volume remains cooler through the thickness, matching intuition regarding localized melting. Using these data, we can also begin to establish lower bounds for the temperature gradient present. As the dimension of the diffraction volume corresponding to the specimen thickness (530 µm) is significantly larger than those defined by the incoming beam (50 and 30 µm), we can assume that the spread of temperature is primarily along the thickness of the specimen. With this being the case, and the hottest portion of the specimen being in the center of the specimen, the lower bound of the temperature gradient can be estimated to be ∼2250 K mm^−1^ (600 K/0.265 mm). With any liquid phase present being significantly hotter, the gradient will be larger, but a lower bound is of value for process design.

### 5.2. Effects of mechanical elastic strain and stress

In Section 4.2, we observed that, in Fig. 9 ▸, all temperature metrics appear to be converging towards predicted temperatures greater than room temperature at the end of data collection. As mentioned, this may be due to accumulation of thermomechanical stress within the specimen. Owing to large thermal gradients in the material during laser melting, mechanical elastic strain and accompanying stress distributions in the material are formed to maintain deformation compatibility. These stresses can become large enough to drive plastic flow and set up residual elastic strain and stress fields that remain in the material even upon cooling (Wang *et al.*, 2017 ▸; Phan *et al.*, 2019 ▸; Bartlett & Li, 2019 ▸). In a cubic alloy such as IN625, tensile hydrostatic stresses and volumetric elastic strains distort the crystal structure, and consequently diffraction patterns, in the same fashion as increased temperature does. However, elastic strains during most mechanical deformation modes have large deviatoric components while thermal strains in cubic materials are solely volumetric. With regards to the diffraction data, heating in the absence of thermal gradients in cubic materials will cause uniform contraction or expansion of the diffraction rings. However, temperature gradients and distributions of thermal strains give rise to elastic strains and mechanical stresses to maintain deformation compatibility that, in turn, will distort diffraction rings into ellipses.

**Figure 11:**
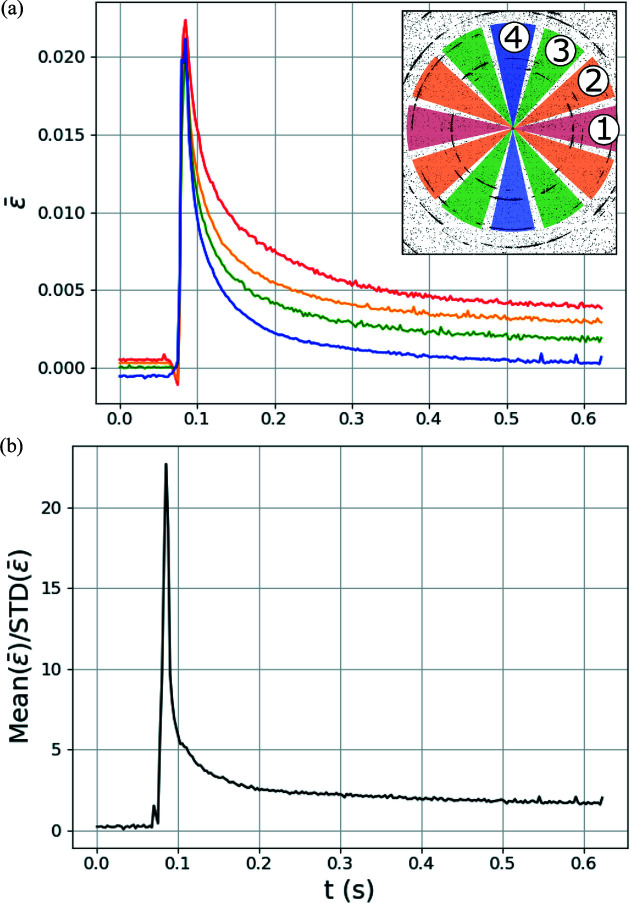
(*a*) Evolution of average lattice strains 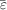 measured from four different regions on the detector (shown in inset) through time *t*. (*b*) Evolution of the ratio of the mean and standard deviation (STD) of the lattice strains from the four different regions.

To explore the role of mechanical elastic strain effects on the temperature predictions, which are not currently included in the laser melting and diffraction simulations, the anisotropy of lattice strain around diffraction rings during the experiment was probed. Fig. 11 ▸(*a*) shows the evolution of average lattice strains from the first three sets of lattice planes from four different azimuthal regions around the detector. These regions are illustrated on a detector image in the inset of Fig. 11 ▸(*a*). Average lattice strains 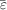 were first found by fitting pseudo-Voigt peaks to the first three sets of lattice planes in each bin [noting that fits are relatively poor in the high-temperature region due to peak splitting, Fig. 6 ▸(*d*)]. Lattice strains from each peak ɛ_*hkl*_ were then determined from fitted peak centers 2θ_*hkl*_ and Bragg angles calculated from the reference lattice parameter 2θ_*hkl*0_: 

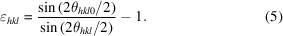

Average lattice strains 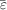 from each region were calculated as an intensity-weighted average of the lattice strains from each peak: 

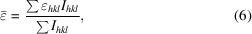

where *I*_*hkl*_ is the fitted maximum intensity of each peak. This averaging of lattice strains from the four different azimuthal regions assumes that the principal strain directions are nominally aligned with the sample edges and the sample coordinate system. The relatively large azimuthal regions were chosen to increase the number of grains contributing to each lattice strain measurement. In Fig. 11 ▸(*a*), as the sample cools, there is a marked deviation in the lattice strains around the detector, reflecting the development of thermomechanical stresses in the specimen containing a tensile stress in the 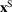 direction. None of the lattice strains in any of the regions become negative, indicating still elevated temperature or a compressive stress along the beam direction 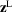 ( 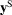 ). The exact internal-stress magnitudes cannot be calculated, but the strains of order 10^−3^ seen in Fig. 11 ▸(*a*) indicate internal stresses on the order of several hundred megapascals as the elastic modulus of AM IN625 is ∼200 GPa (Wang *et al.*, 2016 ▸). Stresses of this magnitude are a significant fraction of the yield strength of the alloy (∼500 MPa; Nguejio *et al.*, 2019 ▸) and may even be sufficient to induce plastic flow. There is also a spread of lattice strains prior to heating that is most likely due to stresses imposed during sample mounting.

To examine an approximate measure of the ratio of volumetric to deviatoric strains caused by thermal expansion and mechanical stresses, respectively, the time evolution of the ratio of the mean and standard deviation of the lattice strains from the four regions is shown in Fig. 11 ▸(*b*). In regions where the ratio is high (>5), the strains are dominated by volumetric (thermal) expansion and the GPR model temperature predictions are more accurate, but as the ratio gets smaller (during cooling), deviatoric (mechanical elastic) strains influence the results to produce inaccurate temperature predictions. This is reflected in the still relatively high temperature metrics determined from the GPR surrogate models in Fig. 10 ▸.

A recent experimental effort (Schmeiser *et al.*, 2021 ▸) has demonstrated that with a large panel area detector capturing full diffraction rings, the effects of mechanical stresses could largely be decoupled from the effects of heating. Keeping this in mind, the method presented in this work could be extended to train GPR surrogate models with diffraction patterns including thermomechanical effects, rather than just thermal effects. The GPR surrogate models would also need to be provided with independent diffraction line profiles from different azimuthal angles around the detector, but this should be readily possible. If successful, low-temperature measurement accuracy will be significantly enhanced.

### 5.3. Melt-pool volume estimation

**Figure 12:**
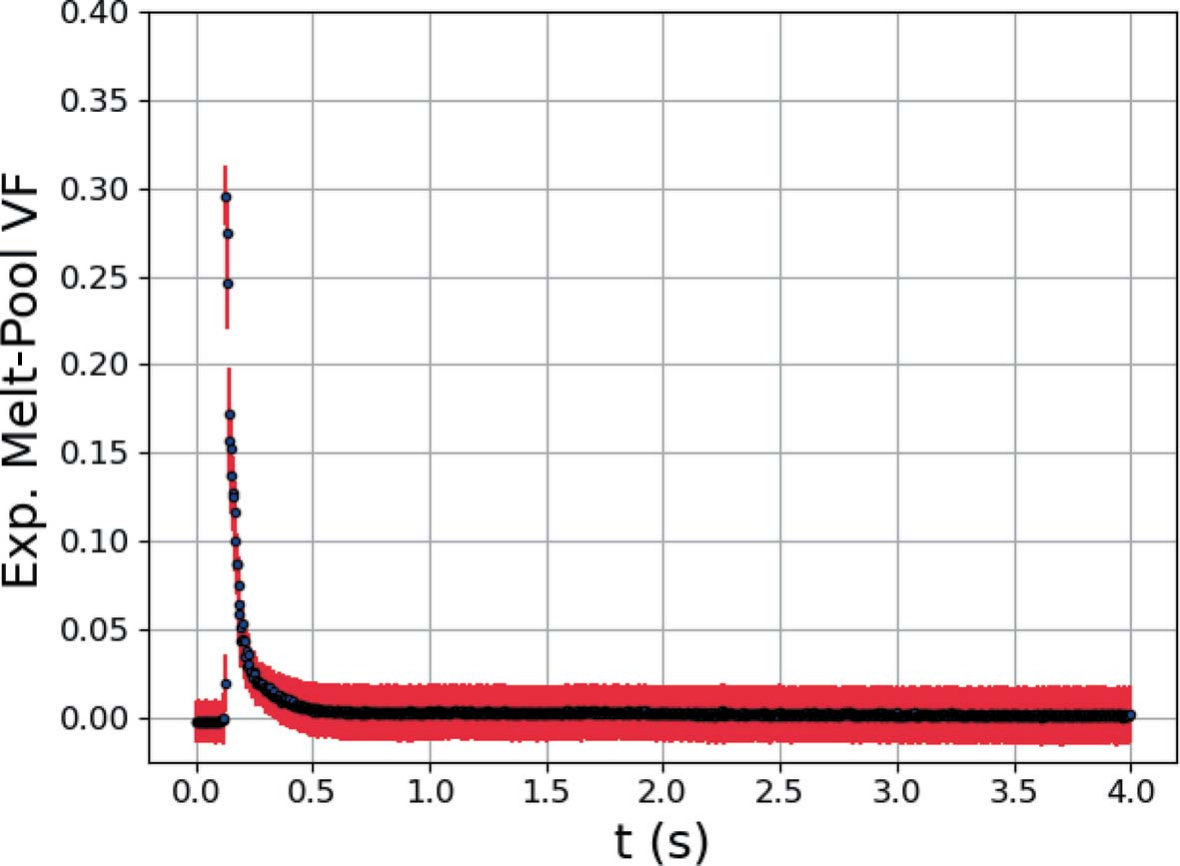
Estimated experimental melt-pool volume fraction (VF) versus time *t*, determined from the experimental diffraction data using a trained GPR surrogate model.

For the extraction of temperature-distribution metrics, the GPR surrogate models that have been presented are primarily learning connections between diffraction peak shape and position with the temperature distributions present in the illuminated diffraction volume. As has been mentioned, when a volume of material melts, that volume will no longer contribute intensity to the measured diffraction peaks. The total integrated intensity will therefore reflect the volume of unmelted alloy and, the converse, the relative volume of the melt pool. To test this idea, a final GPR surrogate model was trained in the same fashion as described above, but trained to connect diffraction line profiles to the volume fraction of the melt pool present. This surrogate model was then provided with the experimental X-ray data, similarly to Section 4.2. Fig. 12 ▸ shows the evolution of the melt-pool volume fraction during the experiment. We can see that the melt-pool volume fraction has a maximum of 0.3 when the laser passes over the diffraction volume. With the relatively large laser-beam size (100 µm) and high power density laser conditions, a melt-pool volume extending through ∼30% of the diffraction volume (150 µm) appears reasonable. While the GPR surrogate model here is utilizing the drop in intensity in the primary diffraction peaks to determine the current melt-pool volume fraction, liquid rings at positions associated with the *n*th nearest-neighbor average atomic distances (pair distribution function) also appear in the data (Waseda & Suzuki, 1972 ▸; Iqbal *et al.*, 2006 ▸). With the appropriate liquid-scattering model in the X-ray scattering simulations (Heinen & Drewitt, 2022 ▸), the accuracy of this GPR surrogate model could be improved. To even further enhance accuracy, the scattering modeling and accompanying surrogate model can be modified to include the effects of background thermal diffuse scattering. While high-speed X-ray radiography has been used extensively in the literature to image melt-pool size, diffraction and the approach presented may provide a complementary means to characterize sub-surface melt-pool dynamics in alloys in addition to samples with relatively little density difference between liquid and solid phases and minimal radiographic imaging contrast.

## 6. Summary and conclusions

For the first time, bulk maximum and minimum temperatures of the solid phase in an engineering alloy (along with temperature-distribution information) have been extracted from *in situ* AM measurements. This was made possible by using high-fidelity mechanistic and supervised machine-learning modeling to determine quantities from the experimental data, as opposed to taking a traditional approach of using experimental data to calibrate the models. The approach consists of training GPR surrogate models using a combination of heat-transfer and fluid-flow modeling and X-ray diffraction modeling. Each surrogate model links diffraction line profiles to metrics describing the temperature distribution present within the diffraction volumes, including maximum, minimum, mean and median temperature. The smallest uncertainties determined from the GPR models are ∼5% for the minimum, median and mean temperatures. In contrast, the largest uncertainty is for the maximum temperature (∼15%), near the solidus temperature. The trained surrogate models were successfully applied to extracting these metrics from *in situ* high-energy synchrotron diffraction data collected during additive manufacturing (laser melting) of a thin wall of Inconel 625. Despite the larger uncertainties from the GPR surrogate model output, temperature-metric accuracy is believed to be greatest in the high-temperature regime and decreases in the low-temperature regime when applied to experimental data, as stress develops and distorts the material present. The *Discussion* [Sec sec5] described future efforts to account for thermomechanical stress formation to increase model accuracy and extend the approach to extract other information about the volume probed, including the volume of the melt pool in the diffraction volume.

## 7. Data and code availability statement

All data used for this work are available upon reasonable request. The Python-based diffraction simulation and GPR training codes are also available upon request. 

## Figures and Tables

**Table 1:** Properties of Inconel 625 used in the heat-transfer and fluid-flow modeling These properties represent the thermo-physical behavior of the alloy and affect the thermal cycles. Here, thermal conductivity and specific heat are taken as temperature dependent and the temperature in kelvin is represented by *T*. The properties were calculated using *JMatPro*.

Physical property	Value
Density (kg m^−3^)	8440
Solidus temperature (K)	1563
Liquidus temperature (K)	1623
Specific heat (J kg^−1^ K^−1^)	360.4 + 0.26*T* − 4 × 10^−5^ *T* ^2^
Thermal conductivity (W m^−1^ K^−1^)	0.56 + 2.9 × 10^−2^ *T* − 7 × 10^−6^ *T* ^2^
Latent heat of fusion (J kg^−1^)	209.2 × 10^3^
Viscosity (kg m^−1^ s^−1^)	5.3 × 10^−3^
Temperature coefficient of surface tension (N m^−1^ K^−1^)	−0.37 × 10^−3^
Surface tension (N m^−1^)	1.82
Absorptivity factor	0.3
Emissivity factor	0.4

**Table 2:** Test matrix showing the nine sets of power–velocity parameters that were used in the thermal simulation

*P* = 100 W	*P* = 100 W	*P* = 100 W
*v* = 0.04 ms^−1^	*v* = 0.05 ms^−1^	*v* = 0.06 ms^−1^

*P* = 120 W	*P* = 120 W	*P* = 120 W
*v* = 0.04 ms^−1^	*v* = 0.05 ms^−1^	*v* = 0.06 ms^−1^

*P* = 140 W	*P* = 140 W	*P* = 140 W
*v* = 0.04 ms^−1^	*v* = 0.05 ms^−1^	*v* = 0.06 ms^−1^
